# High Prevalence of Molecular Markers Associated with Artemisinin, Sulfadoxine, and Pyrimethamine Resistance in Northern Namibia

**DOI:** 10.4269/ajtmh.24-0870

**Published:** 2025-07-31

**Authors:** Lydia Eloff, Andrés Aranda-Díaz, Isobel Routledge, Amy Wesolowski, Mukosha Chisenga, Brighton Mangena, John Chimumbwa, Chadwick Sikaala, Petrina Uusiku, Stark Katokele, Jaishree Raman, Jennifer L. Smith, Davis R. Mumbengegwi

**Affiliations:** ^1^ Department of Biochemistry, Microbiology, and Biotechnology, School of Science, University of Namibia, Windhoek, Namibia;; ^2^ Malaria Operational Research Program, Multidisciplinary Research Services, University of Namibia, Windhoek, Namibia;; ^3^ EPPIcenter Research Program, Department of Medicine, University of California, San Francisco, California, USA;; ^4^ Malaria Elimination Initiative, Institute of Global Health Sciences, University of California, San Francisco, California, USA;; ^5^ Department of Epidemiology, Johns Hopkins University Bloomberg School of Public Health, Baltimore, Maryland, USA;; ^6^ SADC Malaria Elimination Eight Secretariat, Windhoek, Namibia;; ^7^ National Vector-Borne Disease Control Program, Ministry of Health and Social Services, Windhoek, Namibia;; ^8^ Laboratory for Antimalarial Resistance Monitoring and Malaria Operational Research (ARMMOR), Centre of Emerging Zoonotic and Parasitic Diseases, National Institute for Communicable Diseases, Johannesburg, South Africa;; ^9^ Wits Research Institute for Malaria, University of Witwatersrand, Johannesburg, South Africa;; ^10^ UP Institute for Sustainable Malaria Control, University of Pretoria, Pretoria, South Africa;; ^11^ Department of Epidemiology & Biostatistics, University of California, San Francisco, California, USA

## Abstract

Artemisinin-based combination therapies are a cornerstone of Namibia’s efforts to eliminate malaria. Namibia has experienced a greater than 90% reduction in malaria cases since the introduction of these therapies in 2005. However, their efficacy has not been routinely monitored, with malaria outbreaks regularly reported since 2016. The recent emergence of artemisinin partial resistance in Africa has highlighted the role of malaria molecular surveillance in complementing efficacy studies. This cross-sectional genomic surveillance study was nested within Namibia’s routine surveillance system and aimed to determine the prevalence of antimalarial drug resistance markers in northern Namibia. Dried blood spots (DBS) and epidemiological data were collected from patients with confirmed *Plasmodium falciparum* cases who presented at health facilities in the highest malaria-burden regions (Zambezi, Kavango East, Kavango West, Ohangwena, and Omusati) from April to September 2023. Twelve genes associated with resistance to seven antimalarial drugs were genotyped from 264 DBS using multiplexed targeted amplicon sequencing. Multiple *kelch 13* mutations associated with artemisinin partial resistance were identified: the P441L candidate marker was the most abundant, at 33.2%, and the P574L and A675V validated markers were observed in 1.2% of samples. The chloroquine resistance transporter C72/V73/M74I/N75E/K76T haplotype was observed in 1% of samples, whereas the multidrug resistance protein 1 N86 genotype, which is associated with reduced susceptibility to lumefantrine, was found in all samples. Although sulfadoxine–pyrimethamine is not used in Namibia, a high proportion of sulfadoxine–pyrimethamine resistance-associated mutations in the dihydropteroate synthase and dihydrofolate reductase genes were observed. In this study, we underscore the need for routine genomic surveillance to monitor emerging drug resistance markers and call for further research to define their clinical implications.

## INTRODUCTION

Namibia, a low-malaria transmission southern African country targeting elimination, has 1.7 million people (68% of the population) living in its nine endemic regions who are at risk of contracting the disease.^1^ Despite substantial progress in reducing cases from 538,512 in 2001 to 3,404 in 2019, progress has plateaued, with outbreaks regularly occurring, as evidenced by the 12,993 cases reported in 2023 (Ministry of Health and Social Services, personal communication).^2^^,^^3^ Over the last decade, malaria transmission has shifted from north-central Namibia to the northeastern regions of Kavango East, Kavango West, and Zambezi.^4^ Challenges related to malaria elimination include behavioral changes in Anopheline vectors (with *Anopheles arabiensis* being the most common); a concentration of cases in high-risk groups, such as young men and travelers; cross-border population movement driving importation; and asymptomatic and low parasitemia infections.^5^^–^^8^ Although *Plasmodium falciparum* (*P. falciparum*) accounts for the majority of the infections, other species (such as *Plasmodium ovale* [*P. ovale*], *Plasmodium vivax* [*P. vivax*], and *Plasmodium malariae* [*P. malariae*]) have also been reported and pose unique challenges because of their distinct biology and transmission dynamics.^9^ Finally, the potential emergence and spread of antimalarial drug resistance threaten the efficacy of current treatment regimens, underscoring the need for sustained vigilance.^10^

Chloroquine resistance was first detected in Namibia in 1984, with increasing resistance leading to a policy shift in 2005.^11^ Artemisinin-based combination therapies (ACTs), including artemether–lumefantrine and dihydroartemisinin–piperaquine, replaced chloroquine as first- and second-line malaria treatments, respectively.^12^ Although artemisinin partial resistance has not been observed in the region, recent reports of artemisinin partial resistance in sub-Saharan Africa underscore the urgent need for ongoing surveillance and efficacy monitoring.^13^ Without routine surveillance for antimalarial resistance, early warning signs may be missed, thereby risking the increased selection for and spread of resistant parasites during outbreaks and undermining Namibia’s elimination efforts.

Antimalarial efficacy is typically monitored through therapeutic efficacy studies (TESs), which are resource-intensive and impractical in low-transmission settings with highly mobile populations because of difficulties in achieving target sample sizes.^14^ Therefore, in settings with very low transmission, the WHO has recommended adaptations to the TES protocol or the implementation of integrated drug efficacy surveillance (iDES), along with enhanced molecular surveillance.^15^ The routine molecular surveillance of resistance markers, including cost-effective genomic methods, can facilitate the early detection of emerging drug resistance.^15^^–^^17^ Single-nucleotide polymorphisms (SNPs) in the propeller domain of (Kelch 13; coded by the *pfk13* gene) have been found to be associated with delayed parasite clearance in vitro and in vivo after artemisinin treatment, which is a sign of artemisinin partial resistance.^18^^,^^19^ These SNPs include “candidate” (significant clinical or laboratory evidence for the association) and “validated” (both clinical and laboratory evidence) markers.^14^ Multiple markers of artemisinin partial resistance have recently independently emerged in sub-Saharan Africa, including P441L, C469Y/F, R561H, and A675V in East Africa (Uganda, Rwanda, and Tanzania) and R622I in the Horn of Africa (Ethiopia and Eritrea).^20^^–^^25^

Molecular markers associated with resistance to other antimalarial drugs have been identified and include SNPs associated with resistance to sulfadoxine (in the dihydropteroate synthase [*pfdhps*] gene), pyrimethamine (in the dihydrofolate reductase [*pfdhfr*] gene), and chloroquine (in the chloroquine resistance transporter [*pfcrt*] gene).^26^ These markers are important for the assessment of ACT partner and non-front-line drug resistance. Mutations in the multidrug resistance protein 1 (*pfmdr1*) gene modulate chloroquine resistance and are differentially selected for by amodiaquine, lumefantrine, and piperaquine.^27^ Polymorphisms in the ferrodoxine (*pffd*), apicoplast ribosomal protein S10 (*pfarps10*), multidrug resistance protein 2 (*pfmdr2*), and *pfcrt* genes have been described as a genetic background on which *pfk13* mutations are likely to arise.^28^

No *pfk13* mutations, along with a low prevalence of *pfcrt* and *pfmdr1* mutations, were detected in Namibia from 2016 to 2017.^29^ More recent unpublished data from 2019 to 2020 have revealed the *pfk13* P441L mutation among malaria parasites from agricultural workers in the Zambezi region (unpublished data). As part of the multi-country Genomics of Malaria in the Elimination 8 (GenE8) initiative, malaria molecular surveillance was conducted in Angola, Eswatini, Namibia, South Africa, and Zambia, using a targeted amplicon sequencing workflow. Here, we report on antimalarial resistance markers present in samples collected in 16 health facilities along Namibia’s northern border between April and September 2023.

## MATERIALS AND METHODS

### Study site.

This study was conducted across five distinct administrative regions in northern Namibia, which are classified into transmission zones based on predicted incidence thresholds and geographic locations. Zone 1 (moderate transmission) includes the Kavango West, Kavango East, and Zambezi regions, whereas zone 2 (low transmission) includes the Omusati and Ohangwena regions. Zone 3 (very low transmission) and zone 4 (nonreceptive) were not included in this study (Figure 1). Within each transmission zone, malaria incidence varies considerably, with the Zambezi region reporting the highest incidence among the other regions (Table 1).^30^ The regions included in this study border either Angola, Botswana, or Zambia. Transmission is highly seasonal, occurring between December and June, with cases peaking between February and April during the rainy season.^31^ The first-line treatment for uncomplicated malaria is artemether–lumefantrine, accompanied by a single low dose of primaquine for parasitologically confirmed cases. For severe malaria, intravenous or intramuscular artesunate is administered for at least 24 hours until the patient can tolerate oral medication.^11^^,^^12^

**Figure 1.**
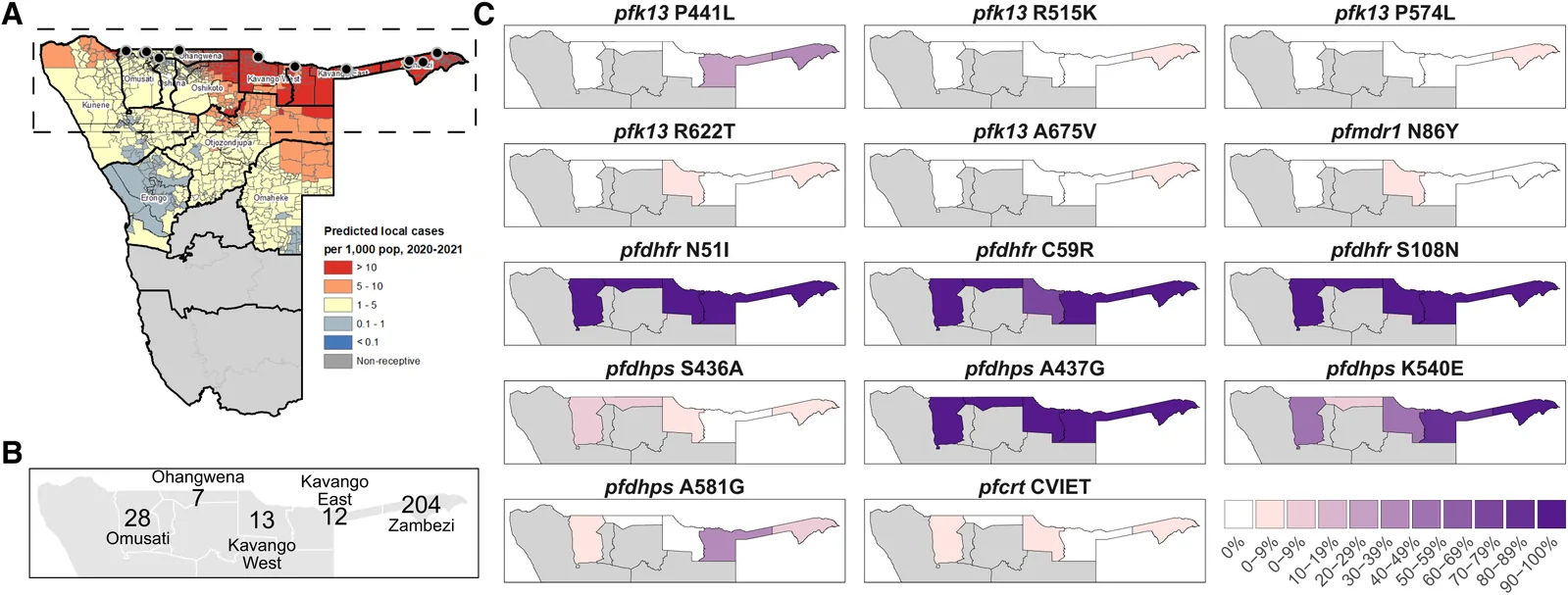
(**A**) Map of Namibia, with risk zones stratified by predicted local cases per 1,000 population in 2020–2021. Data collection was conducted at health facilities indicated by black dots from April to September 2023. (**B**) Number of sequenced samples per region. (**C**) Proportion of samples carrying resistance markers of interest per region. Other mutations and source data can be found in the Supplemental Information.

**Table 1:** Summary of demographic, geographic, and clinical data for individuals with sequenced samples, overall and by region

Characteristic	Overall	Region				
		Omusati	Ohangwena	Kavango West	Kavango East	Zambezi
2023 incidence (cases/1,000 population)	–	1	4	6	5	64
Health facilities	16	4	1	1	5	5
Sequenced samples	264	28 (10.6%)	7 (2.7%)	13 (4.9%)	12 (4.5%)	204 (77.3%)
Country of residence						
Angola	25 (9.5%)	18 (64%)	4 (57%)	3 (23%)	0 (0%)	0 (0%)
Namibia	236 (89%)	10 (36%)	3 (43%)	10 (77%)	12 (100%)	201 (99%)
Zambia	3 (1.1%)	0 (0%)	0 (0%)	0 (0%)	0 (0%)	3 (1.5%)
Age group						
<5	24 (9.1%)	4 (14%)	0 (0%)	2 (15%)	0 (0%)	18 (8.8%)
5–14	88 (33%)	5 (18%)	3 (43%)	4 (31%)	5 (42%)	71 (35%)
15–24	59 (22%)	5 (18%)	1 (14%)	1 (7.7%)	3 (25%)	49 (24%)
25–39	55 (21%)	7 (25%)	1 (14%)	5 (38%)	2 (17%)	40 (20%)
40–59	26 (9.8%)	3 (11%)	2 (29%)	1 (7.7%)	0 (0%)	20 (9.8%)
60+	12 (4.5%)	4 (14%)	0 (0%)	0 (0%)	2 (17%)	6 (2.9%)
Sex						
Female	116 (44%)	16 (57%)	0 (0%)	6 (46%)	2 (17%)	92 (45%)
Male	148 (56%)	12 (43%)	7 (100%)	7 (54%)	10 (83%)	112 (55%)
Travel history						
No travel	243 (93%)	17 (63%)	4 (57%)	12 (100%)	10 (83%)	200 (98%)
Domestic	19 (7.3%)	10 (37%)	3 (43%)	0 (0%)	2 (17%)	4 (2.0%)
International	2	1	0	1	0	0

### Study design and sampling.

This was a cross-sectional study nested within the national surveillance system and conducted as part of the wider GenE8 project. With the sampling strategy, we prioritized representativeness across geographies and transmission strata on the basis of the ecology and incidence of malaria, thereby optimizing resource allocation to cover a significant proportion of the expected cases. In this study, we also targeted a minimum of 50 malaria-positive patients across 16 health facilities along Namibia’s northern border (Figure 1) in the Omusati, Ohangwena, Kavango East, Kavango West, and Zambezi regions. The target sample size (*n* = 800) was intended to be used to 1) estimate a 5% prevalence of a given molecular marker with a precision of 2.0% and 95% confidence and 2) achieve 90% power to detect a prevalence exceeding a value of 2% if the true prevalence is 4.3% using a one-sided binomial test with ɑ = 0.05 and a design effect of 1.5. The achieved sample size of 264 provided a precision of 2.6% for a proportion of 5%. Sampling was conducted through passive case detection during part of a single transmission season (April–September 2023). Symptomatic patients who presented to participating health facilities were tested with a *P. falciparum*/Pan-species Rapid Diagnostic Test (Pf/*Pan* RDT; MERISCREEN Malaria Pf/*Pan* Ag, Meril Diagnostics, Gujarat, India) to detect *P. falciparum* histidine-rich protein 2 and common *Plasmodium* lactate dehydrogenase, according to national guidelines.^12^ All patients who tested positive for malaria and were aged over 2 years were invited to participate. Any participant who had taken malaria prophylaxis or treatment within the preceding 14 days was excluded from the study.

### Sample collection.

Written consent was obtained as described in the Disclosures footnote before recruitment. Consenting participants were interviewed by trained study staff at the point of care in the health facility by using preprogrammed survey tools on encrypted Android tablets that captured information on demographics, clinical symptoms, travel history, occupation, and routine malaria interventions used by the participant or in their residence (including bed net usage, chemoprophylaxis, and indoor residual spraying). Dried blood spots (DBS) collected from finger-prick blood samples were uniquely labeled with barcodes to enable the linking of genomic and survey data, air dried, and stored at –20°C in sealed bags with desiccants until laboratory processing. All participants were treated with the first-line treatment for uncomplicated malaria (artemether–lumefantrine) in line with national guidelines.^12^

### DNA extraction, quantitative real-time polymerase chain reaction (qPCR), and multiplexed amplicon sequencing.

All laboratory procedures were conducted at the National Institute for Communicable Diseases in Johannesburg, South Africa. Only DBS samples that could be linked to their epidemiological data were added to the sequencing workflow. DNA was extracted from a 6 mm hole punch from DBS using the Tween20 (Sigma Aldrich, Saint Louis, MO) - Chelex (Bio-Rad, Hercules, CA) protocol.^32^ A total of 150 *µ*L of 10% Chelex 100 resin in water was used for the final extraction step, and the supernatant was transferred to DNA storage tubes. Parasite density was estimated via qPCR targeting the *var* gene acidic terminal sequence.^33^ Samples with <5 parasites/µL were excluded from further processing. Sequencing libraries were prepared by using an assay based on multiplexed amplicons for drug, diagnostic, diversity, and differentiation using high-throughput targeted resequencing via the MAD^4^HatTeR protocol.^34^ Briefly, a multiplexed PCR using primer pools D1.1, R1.1, and R1.2 was performed with 15 or 20 cycles (for samples with parasitemia ≥10 and <10 parasite/µL, respectively). The multiplexed PCR was followed by bead cleaning, digestion, and an indexing PCR according to the CleanPlex Amplicon Sequencing protocol and using Paragon Genomics (Fremont, CA) reagents. Indexing PCR was performed for 15 cycles by using dual unique indexes. Library quality was checked on a random subset of samples using capillary electrophoresis (Tapestation 4200, using D1000 ScreenTapes and reagents [Agilent Technologies, Santa Clara, CA]). The samples were pooled with volumes inversely proportional to the sample parasitemia, and pools of samples were combined to run on a NextSeq 2000 instrument (Illumina, San Diego, CA) using 150 paired-end reads, with at most 384 libraries per run using a P2 reagent kit.

### Bioinformatics data processing.

The resulting demultiplexed fastq files were processed by using v0.2.1 of a custom bioinformatic pipeline to infer alleles (available on github.com/EPPIcenter/mad4hatter), and further data cleaning was conducted in R (version 4.3.1; R Foundation, Vienna, Austria).^34^ Briefly, reads were demultiplexed into each target and filtered (by quality and length and to remove primer dimers), and amplicon sequences were inferred by using DADA2.^35^ The resulting amplicon sequence variants were then aligned to the reference genome (*P*. *falciparum* 3D7 version 2020-09-01) to filter out nonspecific amplification. To improve precision and specificity, we used the following DADA2 parameters: OMEGA_A = 1e-120 and pool = pseudo.^34^ To select priors for pseudo-pooling in the second round of inference, PSEUDO_ABUNDANCE = 100 and PSEUDO_PREVALENCE = 1,000 were used, which effectively constrained it to alleles observed with more than 100 reads in any sample. Single-nucleotide polymorphisms in target sequences in the *pfk13*, *pfcrt*, *pfmdr1*, *pfmdr2*, *pfdhps*, *pfdhfr*, *pfcoronin*, *pfarpS10*, phosphoinositide-binding protein PX1 (*pfpx1*), *pffd*, exonuclease (*pfexo*), and PF3D7_1322700 genes were assessed. Single-nucleotide polymorphisms contained within the same amplicon are reported as microhaplotypes. Targets in the genes that code for lactate dehydrogenase (*ldh)* in *P. falciparum*, *P. vivax*, *P. ovale*, *P. malariae*, and *Plasmodium knowlesi* were used to detect *Plasmodium* species. To increase precision, samples with less than 10,000 reads, targets with ≤50 reads, and SNPs with within-sample allele frequencies of ≤1% or ≤10 reads were excluded from the analysis.^34^ Non*-falciparum* targets were excluded from sequencing depth summaries. Otherwise, all resulting data were included, including samples with partial coverage of SNPs, resulting in varying total sample numbers for each SNP. A dataset that contains all individual genotypes is available online (DOI:10.5281/zenodo.15855274).

## STATISTICAL ANALYSES

Further data analysis and visualizations were conducted in R (version 4.3.1). Farming and herding occupations were combined into a single category (agricultural workers), whereas all other occupations, excluding minors, students, and the unemployed, were grouped together (other workers). Additionally, to enable statistical analysis, individuals aged 25 years or older were grouped into a single category, and travel reports (within the 2 months before sample collection) were categorized as either international or domestic.

The proportion of samples carrying a mutation (i.e., polyclonal samples with mixed genotypes counted as a carrier of the mutation) was calculated for each health facility and region, as well as the overall proportion, along with their 95% CIs, which were calculated by using the binom.test function in R. Multivariate logistic regression was performed by using a generalized linear mixed-effects model to assess associations between the presence of markers of interest (as a binary outcome) and fixed effects, including age, sex, country of residence, and travel history. A random intercept for each region was incorporated in the model. This analysis was restricted to mutations and variables with sufficient sample size for convergence.

Multilocus haplotypes were constructed for polymorphisms in *pfmdr1* (amino acids 86, 184, and 1,246, targeted by three independent amplicons), and *pfdhps/pfdhfr* (amino acids in *pfdhps*: 436, 437, 540, and 581; amino acids in *pfdhfr*: 51 and 59; targeted by four independent amplicons). Samples with a mixed genotype in one of the amplicons were considered to contain both haplotypes; samples with mixed genotypes in more than two amplicons were considered undetermined.

The complexity of infection (COI) for each sample was calculated as the number of alleles corresponding to the 95th percentile of the allele counts per locus within the sample. The population COI and 95% CIs were obtained via Poisson regression. Allele frequencies were then estimated, considering the COI of each sample, via maximum likelihood estimation. One-dimensional likelihood ratio-based 95% CIs were estimated for the minor allele.

Species-specific *ldh* targets in the panel were used to identify coinfections with non-*falciparum* species. The resulting sequences were used to remove nonspecific amplification and to classify *P*. *ovale* reads into *P*. *ovale curtisi* or *P*. *ovale wallikeri*. Non-*falciparum* species were considered present if the combined reads from all *ldh* targets were >100, with each species contributing >1% of the total *ldh* target reads.

## RESULTS

The 16 selected health facilities reported a total of 1,246 malaria cases during the study period. Of the 278 DBS collected, 263 with complete survey data were subjected to DNA extraction and quantification via quantitative real-time polymerase chain reaction. A total of 11 samples with negative or missing RDT results (three and eight, respectively) were included in the workflow (Supplemental Table 1). A total of 264 samples with parasitemia involving more than 5 parasites/µL were sequenced. A total of 77.3% of sequenced samples came from the Zambezi region, and 56% were collected from male participants (Table 1). Most study participants were Namibian residents (89%), with limited travel outside their habitual residence in the 2 months before enrollment in the study (7.3%). The largest proportion of participants fell within the 5–14 years age group (33%). Aside from minors, frequently reported occupation categories included students and the unemployed.

### Sequencing performance and sequenced sample characteristics.

The overall sequencing depth was high, with a median of 2,312,326 reads per sample (interquartile range [IQR]: 561,791–4,451,199) and 4,051 reads per target (IQR: 1,163–12,919). Of the 264 samples, 253 and 144 had >100 reads in 75% or 95% of the targets, respectively. Overall, parasitemia was high, and successfully sequenced samples had higher parasitemia than unsuccessfully sequenced samples (median: 65,652.4 parasites/*µ*L, *n* = 254, and 8.7 parasites/*µ*L, *n* = 10). More than half (53.5%) of the samples were polyclonal, with a population COI of 1.79 (95% CI: 1.68–1.90, *n* = 254; Figure 2). The COI was not correlated with either parasitemia or sequencing depth (*P* >0.1) but was significantly higher in Kavango West and Omusati than in Zambezi (*P* = 0.03 and 5.4 × 10^−5^, respectively). Non-*falciparum* species, including *P*. *ovale wallikeri*, *P*. *ovale curtisi*, and *P. malariae*, were detected in 5.68% of the samples (Supplemental Table 2). *Plasmodium vivax* was not detected in any samples.

**Figure 2.**
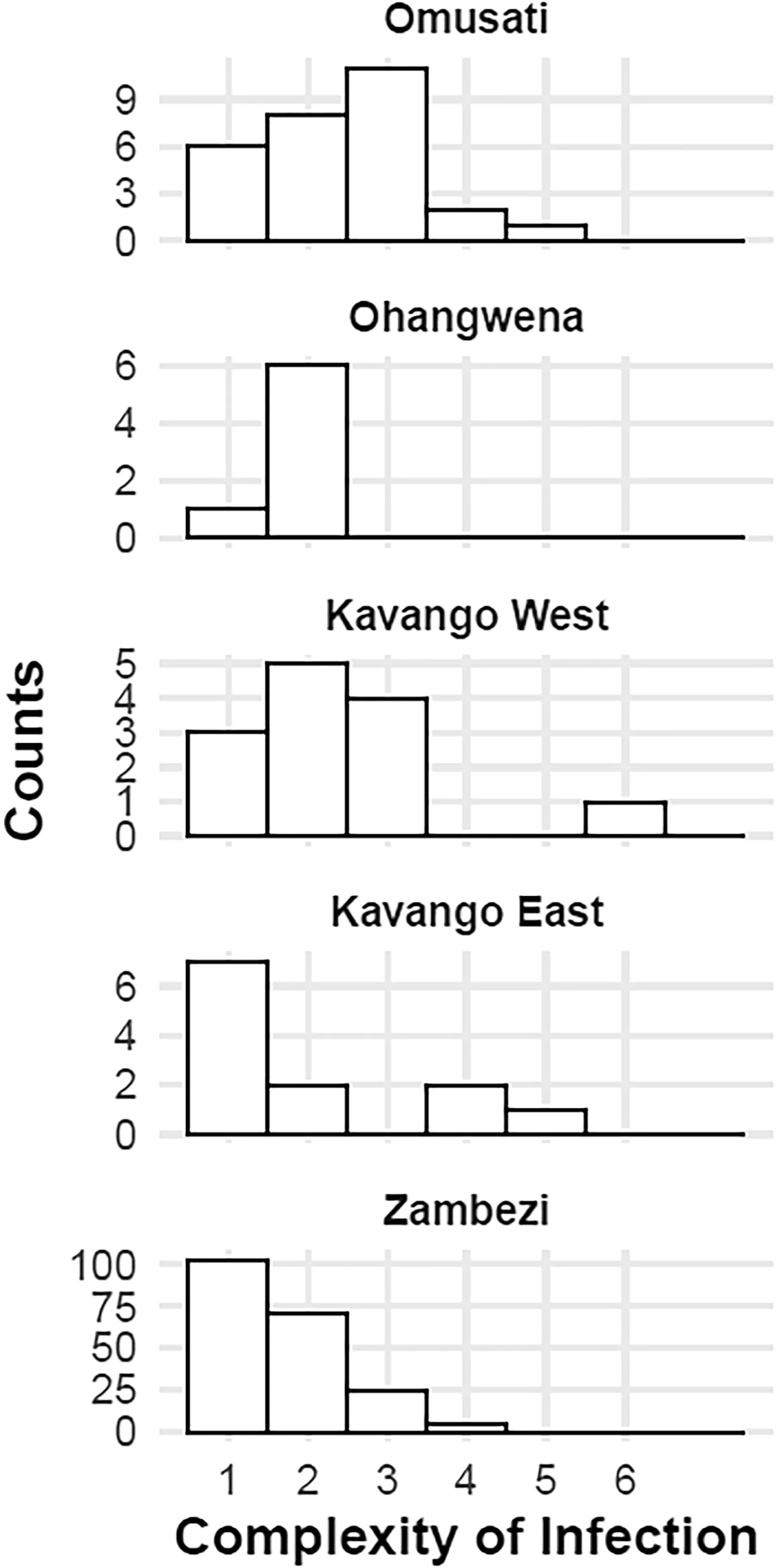
Distribution of the complexity of infection of samples in each region.

### Antimalarial drug resistance molecular markers.

Variants were identified in seven genes: *pfarps10, pfcrt, pfdhfr, pfdhps, pfk13, pfmdr1*, and *pfmdr2*. No variants were detected in *PF3D7_13227700, pfcoronin, pfexo, pffd* and *pfpx1* (Supplemental Table 3).

Several mutations were detected in the *pfk13* gene, including candidate and validated markers of artemisinin partial resistance (Figure 1; Supplemental Tables 3 and 4). The candidate P441L marker was the most abundant mutation detected. Approximately one-third of the samples carried this mutation (85 of 258 samples; 32.9%; 95% CI: 27.2–39.0%), with four samples found in Kavango East (36.4%; 95% CI: 10.9–69.2%) and 81 found in Zambezi (40.1%; 95% CI: 33.3–47.2%). Health facilities within Zambezi reported similar proportions of samples that carried the mutation (Supplemental Table 5). No samples carrying this marker were detected in the western regions of Omusati, Ohangwena, and Kavango West. A total of 45.9% of the infections that carried P441L had a mixed genotype (Supplemental Table 6), and those without a mixed genotype were not clonal. Males (56% of the total sample) were less likely to carry the marker, with an odds ratio of 0.54 (95% CI: 0.39–0.95; Table 2). There was no evidence of an association between P441L and age, residency, or travel history. Two validated markers of artemisinin partial resistance (P574L and A675V), as well as the candidate marker R515K, were detected in the Zambezi region at low levels (Figure 1; Supplemental Tables 4–6). Additional *pfk13* mutations observed in >1% of the samples included a synonymous mutation, A504A (1.6%; 95% CI: 0.4–4.1%) and an uncharacterized mutation, R622T (3.9%; 95% CI: 1.9–7%).

**Table 2:** Multivariate logistic regression results for relevant demographics and drug resistance markers of interest

Characteristic	*pfk13* P441L (85*/247^†^)			*pfdhps* K540E (211*/249^†^)		
	n*/*N*^†^	OR (95% CI)	*P*-Value	n*/*N*^†^	OR (95% CI)	*P*-Value
Residence						
Namibian resident	84/222	Ref.	0.2	197/224	Ref.	0.6
Not a Namibian resident	1/25	0.11 (0.0–2.74)		14/25	0.65 (0.13–3.40)	
Age						
<5	7/22	Ref.	>0.9	16/22	Ref.	0.2
5–14	32/83	1.37 (0.47–3.99)		72/84	2.38 (0.72–7.80)	
15–24	19/58	1.21 (0.39–3.76)		52/58	3.93 (0.98–15.8)	
25+	27/84	1.14 (0.38–3.36)		71/85	2.91 (0.86–9.79)	
Sex						
Female	45/109	Ref.	0.034	92/110	Ref.	0.7
Male	40/138	0.54 (0.30–0.95)		119/139	1.18 (0.53–2.63)	
Travel history						
No travel	82/228	Ref.	0.5	201/230	Ref.	0.13
Domestic	2/10	4.86 (0.36–66.1)		7/10	0.67 (0.11–4.02)	
International	1/9	1.41 (0.10–20.3)		3/9	0.17 (0.03–0.96)	

OR = odds ratio; *pfdhps* = dihydropteroate synthase; pfk13 = *kelch13*.

Only markers present in a sufficient number of samples (empirically estimated at >16%) to ensure model convergence are reported. Results for other markers, including multidrug resistance protein 1 Y184F and multidrug resistance protein 2 I492V, are provided in Supplemental Table 7.

* Number of samples carrying the marker.

^†^ Total number of samples.

The *pfmdr1* N86Y, G182G, and D1246Y mutations were observed in <2% of the samples (Figure 1; Supplemental Tables 4–6). The *pfmdr1* N86 mutation was present in all samples, and only one sample of mixed genotype carried the N86Y mutation. The *pfmdr1* Y184F (53.7%) and *pfmdr2* I492V (52.5%) mutations were present in just over half of the samples and did not show a significant association with any demographic aspects (Supplemental Table 7). The *pfmdr1* N86/Y184/D1246 (NYD) haplotype was carried by the majority of samples (67%), followed by the N86/184F/D1246 (NFD; 50.6%; Supplemental Table 8) haplotype. The *pfcrt* C72/V73/M74I/N75E/K76T (CVIET) microhaplotype, which is associated with chloroquine resistance, was observed in 1.1% of all samples. More than 80% of samples carried pyrimethamine resistance (*pfdhfr* N51I, C59R, and S108N) and sulfadoxine resistance (*pfdhps* A437G and K540E) markers. The quintuple mutant haplotype that contains all these mutations was found in 67% of the samples, whereas the sextuple mutant haplotype that additionally contains the *pfdhps* A581G mutations was found in 8.7% of the samples (Supplemental Table 8). As a single polymorphism, the *pfdhps* A581G mutation was found in 10.8% of the samples and, in all cases, occurred alongside the *pfdhps* K540E mutation within the same microhaplotype. A total of 7.2% of the samples had undetermined *pfdhps*/*pfdhfr* haplotypes. The *pfdhps* S436A mutation was present in 3.4% of the samples, primarily in samples with a *pfdhps* K540 genotype. The *pfdhps* I431V, *pfdhps* A613S/L, and *pfdhfr* I164L markers were not detected in any of the samples. There were no significant associations between the *pfdhps* K540E mutation and any of the demographics (Table 2). However, its proportion was significantly lower in the westernmost region of Omusati compared with the easternmost region of Zambezi (Supplemental Table 4). The observed allele frequencies for all individual markers were lower than the proportion of samples that carried the allele (Supplemental Table 9).

## DISCUSSION

In this study, we report the first published data produced using deep sequencing to characterize antimalarial drug resistance markers in Namibia, a southern African country with low and highly geographically concentrated transmission. The key findings include high levels of markers for artemisinin partial resistance and mutations associated with resistance to sulfadoxine and pyrimethamine. Additionally, the *pfmdr1* N86 genotype, which is associated with reduced susceptibility to lumefantrine, was detected in all samples.^36^^–^^39^ A candidate marker of artemisinin partial resistance, the *pfk13* P441L mutation, was found in a high proportion of the samples, mainly in the Zambezi region (40.1%). This region is in the vicinity of four other countries: Angola, Botswana, Zambia, and Zimbabwe. Approximately half of the infections were polyclonal, with the highest COI found in the lowest-transmission region, Omusati. The genomic assay also revealed a low proportion of coinfections with *P*. *malariae*, *P*. *ovale curtisi*, and *P*. *ovale wallikeri*, as well as no coinfections with *P*. *vivax*.

In this study, we identified both “candidate” and “validated” *pfk13* markers for artemisinin partial resistance in Namibia. The candidate marker *pfk13* P441L mutation, which was first found in the Greater Mekong subregion, has been reported in western Uganda (up to 23% in one site) and at a very low prevalence in Central Africa.^22^^,^^40^ This marker has been associated with delayed clearance in the Greater Mekong subregion; however, in a study with very low sample sizes from that region, it showed no increased ring survival ex vivo.^41^^,^^42^ Its clinical efficacy and phenotypical effects in African parasites remain unassessed.^13^ Clinical or laboratory evidence for the other low-level markers of artemisinin partial resistance found in this study has been reported in Africa; these include the rare markers P574L, R515K, and A675V, which have shown a notable increase in Uganda.^21^^,^^22^^,^^43^ Notably, the R622T mutation, which was observed in 3.9% of the samples, occurs at the same amino acid position as the validated marker R622I, causing delayed clearance in the Horn of Africa and suggesting a potential similar phenotype for the R622T marker.^24^

There was a distinct variation in the distribution of mutations between regions in northern Namibia. Although the Zambezi region had a higher proportion of samples with any *pfk13* mutation, there were limited samples from other regions to generate reliable estimates. Our results did not reveal a significant association between residence in Namibia or travel history and P441L carriage, and data collection occurred late in the transmission season. Nevertheless, most samples corresponded to Namibian residents and participants who did not report travel (84 and 82 of 85 P441L carriers, respectively), and these infections were not clonal, suggesting that multiple parasites carrying the mutation are sustained by local transmission. High parasite connectivity among Namibia, Angola, and Zambia, driven by human mobility, suggests that this mutation is likely to be found in neighboring countries.^6^^,^^7^^,^^44^ The only significant association observed with P441L carriage, although with a wide CI, was a lower likelihood among males. This may reflect unmeasured or misreported factors, such as travel history, and warrants further investigation given the potential implications for targeted interventions to prevent further selection and limit the spread of mutations. Sex-based differences in exposure to P441L hotspots, influenced by work, caregiving, treatment-seeking behaviors, and immunity, especially in younger and pregnant women who are more vulnerable to malaria, could impact parasite clearance, density, and resistance selection.^45^^,^^46^ However, the limited sample size and scope of this study constrained our ability to clarify this relationship. Sample sizes were not substantial enough to identify associations for other *pfk13* mutations, although all participants carrying them were residents who reported no travel. None of the markers described in this study were identified in a survey conducted in the Zambezi region in 2017; however, unpublished data from 2020, along with a recent study from southern Zambia, revealed the presence of the P441L marker, which could indicate cross-border parasite movement.^29^^,^^47^^,^^48^ The interplay among transmission intensity, host immunity, pharmacokinetics, drug pressure, and parasite physiology plays a critical role in shaping the emergence and propagation of drug resistance.^49^ Historically, chloroquine resistance first developed in areas of low malaria transmission, and antifolate resistance has been observed to spread more swiftly in low-transmission regions compared with those with high transmission.^49^ Regions that have experienced the emergence of artemisinin partial resistance are either low-transmission areas or areas that experienced an upsurge in cases.^22^^,^^50^ In Namibia, which is a low-transmission setting, multiple factors may have contributed to the selection of and increase in artemisinin partial resistance markers to the levels described in this study. Recent epidemics may have driven selection by exposing nonimmune populations to malaria, increasing treatment-seeking behavior, and consequently, increasing drug pressure.^13^^,^^22^ Modeling analyses have revealed that the risk of selecting for drug-resistant parasites is high in Namibia, given its low incidence and high artemether–lumefantrine usage.^51^ Lower population immunity due to reduced exposure could also have limited the hosts’ ability to clear drug-resistant parasites despite potential fitness costs.^13^ Additionally, low transmission is typically associated with lower polyclonality (85.3% of the infections in Zambezi had a COI ≤2), reducing within-host parasite competition.^13^^,^^26^ Despite limited sample sizes, the COI was higher in Omusati, a region with very low transmission. This difference likely reflects variations in importation dynamics and seasonal transmission patterns. Omusati, along with the Ohangwena and the Kavango regions, experiences cross-border malaria transmission from Angola, which is a high-burden country.^6^^,^^52^ In contrast, Zambezi’s transmission appears more localized, with lower COI likely driven by the predominance of fewer parasite strains and minimal travel history among residents (98% had not traveled; 99% reported Namibian residency). In Omusati, however, 37% of individuals reported domestic travel history, and 64% identified themselves as Angolan residents, highlighting the role of cross-border transmission. These findings aligned with those of a previous study that revealed lower COI in the Zambezi region.^6^ Environmental factors may also play a role because Zambezi’s higher annual rainfall and permanent water bodies sustain endemic transmission, whereas Omusati’s malaria burden is characterized by seasonal peaks, which are likely driven by imported cases. Other potential contributing factors include increased presumptive treatment due to false positive RDT results and the use of artemisinin-based antimalarials for targeted mass drug administration in Namibia and Zambia, leading to increased drug pressure.^53^^–^^55^ Retrospective studies are necessary to describe the emergence dynamics of these markers and to shed light on the contribution of some of these factors at the time the markers propagated.

Although artemisinin partial resistance may not lead to ACT failures due to the action of the partner drugs, it threatens the efficacy of intravenous artesunate monotherapies used to treat severe malaria.^13^ The reduced efficacy of the artemisinin component also puts additional pressure on the partner drug and could lead to ACT failure in the presence of resistance to the partner drug.^56^ Ensuring the use of efficacious partner drugs is therefore critical. No evidence of full lumefantrine resistance has been documented in Africa, but the high prevalence of the *pfmdr1* N86 and *pfcrt* K76 genotypes has been previously shown to be selected for by lumefantrine use and may be linked to a modest reduction in lumefantrine susceptibility.^15^^,^^36^^–^^39^ Conversely, the *pfcrt* 72–76 CVIET microhaplotype, along with the *pfmdr1* N86Y mutation, are associated with resistance to chloroquine and, to a lesser extent, amodiaquine.^57^ These differences in susceptibility may explain the differential selection of the *pfmdr1* NFD and 86Y/Y184/1246Y haplotypes by artemether–lumefantrine and artesunate–amodiaquine use, respectively.^27^ Our data show that the *pfmdr1* N86 (mostly in the NFD and NYD haplotypes) and *pfcrt* K76 mutations are ubiquitous. These patterns are likely shaped by the discontinuation of chloroquine use in Namibia after resistance was detected in 1984, as well as the heavy reliance on artemether–lumefantrine.^11^ No mutations in *pfcrt* associated with piperaquine resistance were detected.

Sulfadoxine–pyrimethamine (SP) resistance is driven by accumulated mutations in *pfdhps* and *pfdhfr*.^27^^,^^58^ The quintuple mutant haplotype is associated with treatment resistance, and the sextuple mutant haplotype confers even higher levels of resistance.^59^ The quintuple mutant haplotype was found in a majority of the samples, and its hallmark mutation, *pfdhps* K540E, was found at very high levels. The sextuple mutant haplotype that contains *pfdhps* A581G was observed in approximately one-tenth of the samples. Other sextuple mutations, including *pfdhps* A613S/T and *pfdhfr* I164L, were absent. These results are notable because SP use was discontinued in Namibia after the detection of resistance in 2004.^11^ However, its use for chemoprevention in neighboring Angola, Zambia, and Zimbabwe may sustain the selection of these markers in this highly interconnected region.^6^ Both Angola and Zambia have reported a high prevalence of SP resistance mutations.^48^^,^^60^ Despite Namibia discontinuing SP use, the presence of these mutations detected in the country could be indicative of cross-border parasite movement.^6^ Similar to SP, cotrimoxazole, which is widely used for prophylaxis in HIV-infected individuals, targets the folate biosynthesis pathway in *P*. *falciparum*. Thus, the use of cotrimoxazole may contribute to the high levels of SP resistance markers found in this study.^61^ Although this finding does not have implications for Namibia’s case management because the country does not use SP, the high prevalence of SP resistance markers observed in this study raises concerns for neighboring countries that continue to use it. Research indicates that SP remains effective for intermittent preventive treatment during pregnancy (IPTp), thereby reducing neonatal mortality, improving birth outcomes, and serving as a prophylactic measure against malaria.^62^^,^^63^ Nonetheless, additional clinical studies and policy assessments are necessary in countries that depend on SP for IPTp and malaria chemoprophylaxis.

It is important to note that sample collection for the study commenced midway through the malaria season because of administrative delays; this is a limitation because not only were sample sizes lower but the skewed temporal distribution of collection may have impacted study findings. This limited statistical inference and the use of regression models for risk factors and precluded further investigations around importation and transmission patterns early in the season. Sample sizes were higher in the Zambezi region, leading to a higher degree of confidence in the representativeness of findings within the region. Samples were collected exclusively from symptomatic patients at health facilities, potentially underrepresenting parasite populations in asymptomatic infections, recent travelers, and users of traditional and other medicines. Short amplicon sequencing presents challenges in phasing SNPs to construct multilocus haplotypes; to mitigate biases from ambiguous polyclonal samples, we predominantly focused our analysis on individual SNPs. Finally, the finding of non-*falciparum* species in Namibia requires further validation using other methods, given that the assay’s sensitivity and specificity are not well-characterized for this particular application.

Achieving malaria elimination in Namibia requires continuous, effective interventions, yet the threat of antimalarial drug resistance poses a challenge. This study highlights a high prevalence of resistance markers, underscoring the urgent need for further research to evaluate their phenotypic and clinical impacts, including the efficacy of ACTs. The findings also emphasize the importance of molecular surveillance in detecting emerging threats, advocating for the routine, geographically representative monitoring of known, novel, and uncharacterized mutations. To sustain progress, future efforts should focus on strengthening health systems, improving data collection, and fostering community engagement, thereby ensuring the long-term success of malaria control and eventual elimination.

## Supplemental Materials

10.4269/ajtmh.24-0870 Supplemental Materials
